# Artesunate suppresses tumor growth and induces apoptosis through the modulation of multiple oncogenic cascades in a chronic myeloid leukemia xenograft mouse model

**DOI:** 10.18632/oncotarget.3004

**Published:** 2015-02-11

**Authors:** Chulwon Kim, Jong Hyun Lee, Sung-Hoon Kim, Gautam Sethi, Kwang Seok Ahn

**Affiliations:** ^1^ College of Korean Medicine, Kyung Hee University, Seoul 130-701, Republic of Korea; ^2^ Department of Pharmacology, Yong Loo Lin School of Medicine, National University of Singapore, 117597, Singapore

**Keywords:** Artesunate, STAT5, CREB, MAPK, CML

## Abstract

Artesunate (ART), a semi-synthetic derivative of artemisinin, is one of the most commonly used anti-malarial drugs. Also, ART possesses anticancer potential albeit through incompletely understood molecular mechanism(s). Here, the effect of ART on various protein kinases, associated gene products, cellular response, and apoptosis was investigated. The *in vivo* effect of ART on the growth of human CML xenograft tumors in athymic nu/nu mice was also examined. In our preliminary experiments, we first observed that phosphorylation of p38, ERK, CREB, Chk-2, STAT5, and RSK proteins were suppressed upon ART exposure. Interestingly, ART induced the expression of SOCS-1 protein and depletion of SOCS-1 using siRNA abrogated the STAT5 inhibitory effect of the drug. Also various dephosphorylations caused by ART led to the suppression of various survival gene products and induced apoptosis through caspase-3 activation. Moreover, ART also substantially potentiated the apoptosis induced by chemotherapeutic agents. Finally, when administered intraperitoneally, ART inhibited p38, ERK, STAT5, and CREB activation in tumor tissues and the growth of human CML xenograft tumors in mice without exhibiting any significant adverse effects. Overall, our results suggest that ART exerts its anti-proliferative and pro-apoptotic effects through suppression of multiple signaling cascades in CML both *in vitro* and *in vivo*.

## INTRODUCTION

Abundant evidence has demonstrated that several phytochemicals found in medicinal herbs exert anti-tumorigenic activities by inducing apoptosis in cancer cells. Plant-derived natural products as well as semisynthetic and synthetic analogs contribute significantly to cancer chemotherapy because they can induce apoptosis in malignant tumor cells while exerting low toxicity towards normal cells and thus producing lower adverse effects [1, 2]. Many recent preclinical and clinical studies have indicated that targeting multiple signaling pathways against neoplastic cells could increase patient survival and might reduce the emergence of cells that are resistant to monotherapy [3]. Thus identification of pharmacological agents that can target diverse oncogenic pathways in tumor cells can form the basis of novel therapy for cancer patients.

Artesunate (ART), extracted from *Artemisiaannua* L. (Sweet Wormwood, *qinghao*), has been used in traditional Chinese medicine to treat fever and chills [4]. Although the active constituent of the plant, artemisinin, has been identified as an anti-malarial sesquiterpene, its derivative ART is deemed more suitable for drug development due to its aqueous solubility. ART is not only cytotoxic towards cancer cell lines *in vitro*, but also exerts significant antitumor activities against human xenograft tumors in nude mice [5–8]. ART has also shown significant anti-proliferative and pro-apoptotic effects against leukemia [9–11], liver cancer [12, 13], oral cancer [14], breast cancer [15], cervical cancer [16], and gastric cancer [17]. Additionally, ART has also been reported to induce apoptosis through inhibition of hyperactive Wnt/beta-catenin [6], NF-kappaB [18], and PI3K/Akt [16] signaling pathways, by targeting iron-catalyzed lysosomal reactive oxygen species [15] activating Bak-mediated caspase-independent intrinsic pathway [19], and promoting cell oncosis [17]. Although several molecular mechanisms as discussed above have been described to account for the potent antitumor activities exerted by ART, its potential effect on p38/ERK/STAT5/CREB signal transduction pathways in human CML cells has never been investigated before.

Several studies have indicated that mitogen-activated protein kinases (MAPKs) such as ERK, p38 kinase, and JNK pathways regulate cellular proliferation, apoptosis, and differentiation [20]. Especially, the p38 kinase and ERK activation are known to contribute to anti-apoptosis by mediating cell proliferation and survival [21]. Besides, the signal transducers and activators of transcription 5 (STAT5) plays a critical role in Breakpoint Cluster Region-Abelson 1 (BCR-ABL1)-driven neoplasias. STAT5 is an essential component in the signaling network that maintains the survival and growth of chronic myeloid leukemia (CML) cells [22]. STAT5 belongs to the STAT gene family [23]. The two highly homologous (98%) isoforms of STAT5, STAT5a and STAT5b [23], act as both cytoplasmic signaling proteins and nuclear transcription factors. STAT5 becomes active by phosphorylation of a specific tyrosine residue in the carboxy-terminal domain [23], homo- or heterodimerizes, and translocates to the nucleus where it binds to the target gene promoters [23]. Previous data has demonstrated that the MAPK pathway is required for the full activation of one of the STAT5 isoforms (STAT5a) [24] and ERK can directly interact with STAT5a [25]. Besides, the dysregulation of the STAT5 signaling pathway is closely associated with oncogenesis and leukemogenesis [26]. STAT5 has been shown to be constitutively activated in several forms of lymphoid, myeloid, and erythroid leukemias [27–29]. Indeed, the introduction of constitutively active STAT5 mutants into hematopoietic cells is sufficient to induce multilineage leukemia in mice [30]. Also, cAMP-regulatory element-binding protein (CREB) is a 43 kDa basic/leucine zipper (bZIP) transcription factor that is overexpressed and constitutively phosphorylated in a number of different human cancers [31]. The downregulation of CREB can inhibit proliferation due to decreasing cells in S phase in the TF-1 and K562 cells showing that CREB indeed can act as a proto-oncogene that potentially contributes to leukemogenesis [32].

The purpose of this study was to investigate the role of ART in inducing human CML cells apoptosis and elucidate its underlying molecular mechanisms. We particularly aimed to determine whether the modulation of p38/ERK/STAT5/CREB signaling pathways by ART can play a critical role in mediating its antitumor effects in human CML cells and xenograft mouse model.

## RESULTS

### ART decreases phosphorylation levels of various kinases in KBM-5 cells

**Figure 1:**
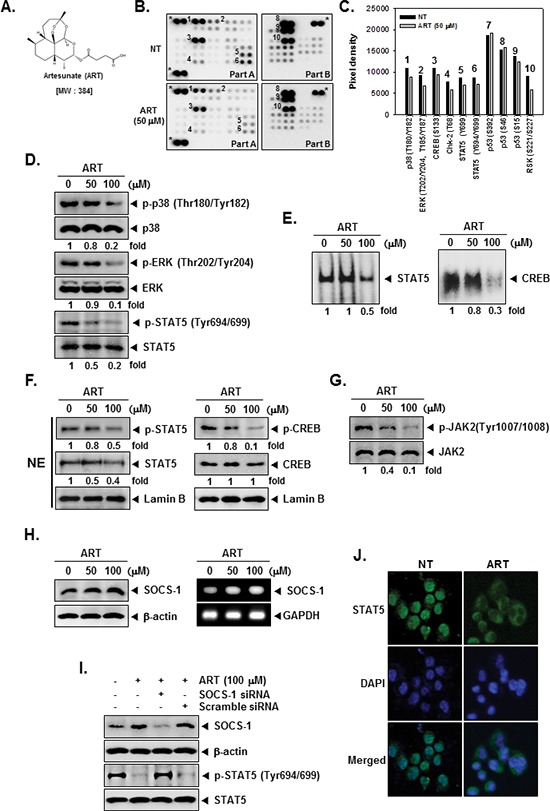
The Human Phospho-Kinase Array detection kit reveals that ART suppresses phosphorylation of p38, ERK, STAT5, and CREB in KBM-5 cells **(A)** The chemical structure of Artesunate (ART). **(B)** KBM-5 human myeloid leukemia cells (1 × 10^7^ cells/well) were either untreated or treated with 50 μM of ART for 4 h. Parts A and B of the array were each incubated with 300 μg of cell lysate. Arrays were done according to the manufacturer's protocols using Human Phospho-Kinase Array Kit (R&D Systems, Minneapolis, MN), and array images are shown. **(C)** Array profiles created by quantifying the mean spot pixel densities are shown. Graphs represent spot intensities of indicated proteins. **(D)** After KBM-5 cells (1 × 10^6^ cells/well) were seeded onto 6-well plates, they were treated with various indicated concentrations of ART for 4 h. Thereafter, equal amounts of lysates were analyzed by Western blot analysis using antibodies against p-p38, p38, p-ERK, ERK, p-STAT5 and STAT5. **(E)** ART suppresses STAT5 and CREB-DNA binding activity. KBM-5 cells (1 × 10^6^ cells/well) were treated with various indicated concentrations of ART for 4 h, analyzed for nuclear STAT5 and CREB levels by EMSA. **(F)** KBM-5 cells (1 × 10^6^ cells/well) were treated with various indicated concentrations of ART for 4 h. After that, nuclear proteins were extract, equal amounts of lysates were analyzed by Western blot analysis using antibodies against p-STAT5, STAT5, p-CREB, and CREB. **(G)** After KBM-5 cells (1 × 10^6^ cells/well) were seeded onto 6-well plates, they were treated with various indicated concentrations of ART for 4 h. Thereafter, equal amounts of lysates were analyzed by Western blot analysis using antibodies against p-JAK2, and JAK2. **(H)** KBM-5 cells (1 × 10^6^ cells/well) were treated with various indicated concentrations of ART for 4 h, after which whole-cell extracts were prepared and 10 μg portions of those extracts were resolved on 8% SDS-PAGE, electrotransferred onto nitrocellulose membranes, and probed for SOCS-1 antibody. The same blots were stripped and reprobed with β-actin antibody to verify equal protein loading (H, *left panels*). KBM-5 cells (1 × 10^6^ cells/well) were treated with various indicated concentrations of ART for 4 h, and total RNA was extracted and examined for expression of SOCS-1 by RT-PCR. GAPDH was used as an internal control to show equal RNA loading (H, *right panels*). **(I)** Effect of SOCS-1 knockdown on ART induced expression of SOCS-1. KBM-5 cells were transfected with either SOCS-1 siRNA or scrambled siRNA (50 nM). After 48 h, cells were treated with 100 μM ART for 4 h and whole-cell extracts were subjected to Western blot analysis. **(J)** ART causes the inhibition of translocation of STAT5 to the nucleus. After 4 h of ART treatment, the cells were fixed and permeabilized. STAT5 (green) was immunostained with mouse anti-STAT5 followed by FITC-conjugated secondary antibodies and the nuclei (blue) were stained with DAPI. The third panels show the merged images of the first and second panels. The results shown are representative of two independent experiments.

The structure of ART is shown in Figure 1A. To examine the effects of ART on intracellular signaling, we screened the phosphorylation status of multiple cellular kinases in human CML KBM-5, using the human phospho-kinase antibody array (Figure 1B and 1C). Activated p38, ERK, CREB, and STAT5 was strongly expressed in non-treated cells. ART treated cells resulted in a decrease in phosphorylated p38, ERK, CREB, and STAT5 levels. Other kinases or phospho-proteins modulated upon ART treatment included: Chk-2, p53, and RSK. The kinases known to phosphorylate CREB, which are linked closely to tumor progression and metastasis [33], include mitogen-activated protein kinases (MAPK) [34]. In addition, STAT5 activation pathways have been closely linked with the proliferation, anti-apoptosis, and chemoresistance of tumors [35]. Therefore, in the next set of experiments, we focused on investigating the potential effect of ART on the CREB and STAT5 signaling pathways in KBM-5 cells.

### ART inhibits phosphorylation of p38, ERK, and STAT5 in KBM-5 cells

Data obtained from our phospho-kinase antibody array studies was further confirmed by Western blot analysis. Cells were treated with indicated concentrations of ART for 4 h. ART suppressed the phosphorylation of p38, ERK, and STAT5 in a concentration-dependent manner in KBM-5 cells and had no effect on the expression of total p38, ERK, STAT5 proteins (Figure 1D).

### ART inhibits binding of STAT5 and CREB to the DNA

Because tyrosine phosphorylation causes the dimerization of STATs and their translocation to the nucleus, where they bind to DNA and regulate gene transcription [36], and also of CREB related transcription factor which binds CRE and heterodimerizes with CREB in nucleus [32], we determined whether ART suppresses the DNA binding activities of both STAT5 and CREB in tumor cells. EMSA analysis of nuclear extracts prepared from KBM-5 cells showed that ART inhibited STAT5- and CREB-DNA binding activities in a dose-dependent manner (Figure 1E). These results show that ART abrogates the DNA binding ability of both STAT5 and CREB proteins.

### ART suppresses phosphorylation of STAT5 and CREB in nuclei

Because STAT5 have shown to play a role in tumor development and progression [30] and the phosphorylation of CREB is linked closely with tumor progression and metastasis [33], we next tested the effect of ART on phosphorylation of STAT5 and CREB in nuclei. As shown by Western blot analysis in Figure 1F, *left panels*, ART inhibited phosphorylation of STAT5 and STAT5 expression in nuclei (*first and second panels*). Likewise, ART suppressed phosphorylation of CREB in a dose-dependent manner, and ART had no effect on CREB protein (Figure 1F, *right*, *first and second panels*).

### ART suppresses constitutive activation of JAK2

Signal transducer and activator of transcription has been reported to be activated by the soluble tyrosine kinases of the Janus family (JAK) [37]. Because JAK2 is the main kinase involved, we examined the effect of ART on JAK2 activation in CML cells. As shown in Figure 1G, JAK2 was constitutively active in KBM-5 cells and the treatment with ART clearly suppressed this phosphorylation in a concentration-dependent manner. The levels of total JAK2 remained unchanged under the similar conditions (Figure 1G, *second panel* ).

### ART induces the expression of SOCS-1 in KBM-5 cells

Suppressors of cytokine signaling (SOCS) are transcriptional targets of activated STAT proteins that negatively control STAT signaling. Thus, we examined whether ART modulates the expression of SOCS-1, which can act as a negative regulator of the JAK/STAT signaling pathway [38]. As shown in Figure 1H, *left panels*, ART led to an increased expression of SOCS-1 at the protein level. ART also enhanced mRNA level of SOCS-1 in a dose dependent manner in KBM-5 cells (Figure 1H, *right panels*).

### SOCS-1 siRNA down-regulate the expression of SOCS-1 and reverses the inhibition of STAT5 activation by ART

We determined whether the suppression of SOCS-1 expression by siRNA would abrogate the inhibitory effect of ART on STAT5 activation. Western blotting showed that ART-induced SOCS-1 expression was effectively abolished in the cells treated with SOCS-1 siRNA; whereas treatment with scrambled siRNA had no effect (Figure 1I, *first panel*). We also found that ART failed to suppress STAT5 activation in cells treated with SOCS-1 siRNA (Figure 1I, *third panel*). These siRNA results corroborate with our earlier evidence on the critical role of SOCS-1 in the suppression of STAT5 phosphorylation by ART.

### ART reduces nuclear pool of STAT5 in tumor cells

Because the active dimer of STAT5 is capable of translocating to the nucleus and inducing transcription of specific target genes [39], we determined whether ART suppresses the nuclear translocation of STAT5. Immunocytochemistry (Figure 1J) clearly demonstrated that ART reduced the translocation of STAT5 into the nucleus in KBM-5 cells.

### ART causes the accumulation of the cells in the sub-G1 phase of the cell cycle in a variety of human tumor cells

**Figure 2:**
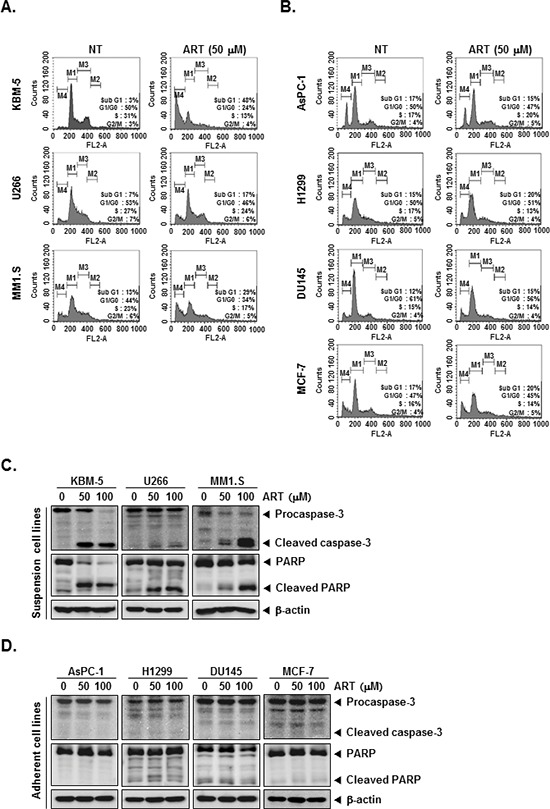
ART induces apoptosis by PARP cleavage through activation of caspase-3 **(A)** and **(B)** After KBM-5, U266, MM1.S, AsPC-1, H1299, DU145, and MCF-7 cells (1 × 10^6^ cells/well) were seeded onto 6-well plates, they were either untreated or treated with 50 μM of ART for 24 h. Then, the cells were fixed and analyzed using a flow cytometry. **(C)** and **(D)** Various tumor cells (1 × 10^6^ cells/well) were treated with indicated concentrations of ART for 24 h. Thereafter, equal amounts of lysates were analyzed by Western blot analysis using antibodies against caspase-3 and PARP. The same blots were stripped and reprobed with β-actin antibody to verify equal protein loading.

We set out to determine the effect of ART on cell cycle distribution in KBM-5, U266, MM1.S, AsPC-1, H1299, DU145, and MCF-7 cells. After treatment each cell lines for 24 h, ART-induced an increased accumulation of cell population on sub-G1 phase in KBM-5, U266, MM1.S cell lines, which is indicative of apoptosis (Figure 2A). Distinctively, ART had no effect on AsPC-1, H1299, DU145, and MCF-7 cell lines, which may be indicative of its cell-type specificity (Figure 2B).

### ART activates caspase-3 and causes PARP cleavage in KBM-5, U266, and MM1.S cells

Cells were treated with indicated concentrations of ART for the 24 h, and then examined for caspase activation by Western blot analysis using specific antibodies. We found a dose-dependent activation of caspase-3 by ART in suspension cell lines (Figure 2C, *first panels*). Activation of downstream caspase-3 led to the cleavage of a 116 kDa PARP protein into 87 kDa fragments (Figure 2C, *Second panels*). In adherent cell lines, on the other hand, ART had no effect on activation of caspase-3 and cleavage of PARP protein (Figure 2D). Taken together, these results strongly suggest that ART induces caspase-3-dependent apoptosis in KBM-5, U266, and MM1.S cell, but not AsPC-1, H1299, DU145, and MCF-7 cells.

### ART down-regulates expression of various proteins involved in apoptosis

**Figure 3:**
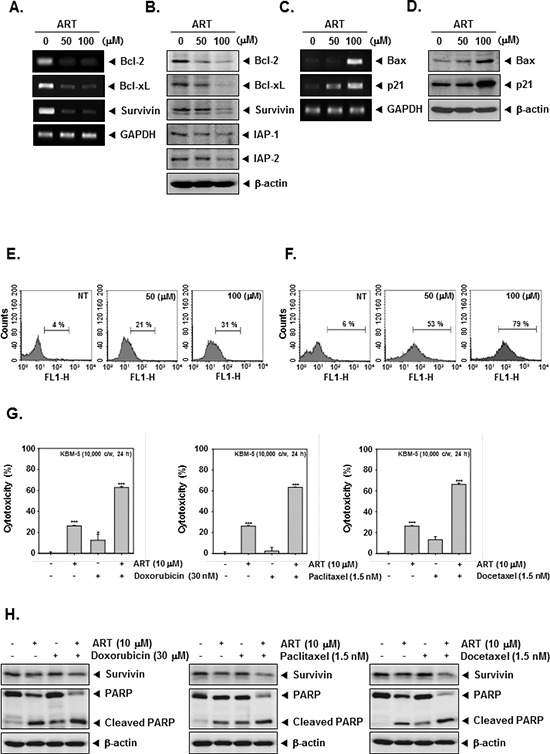
ART and chemotherapeutic agents induce apoptosis **(A)** Inhibition of bcl-2, bcl-xL, and survivin mRNA expressions by ART in KBM-5 cells. Cells (1 × 10^6^ cells/well) were treated with indicated concentrations of ART for 24 h. Total RNA was isolated, bcl-2, bcl-xL, and survivin mRNA expressions were examined by RT-PCR analysis. GAPDH was performed to control for a similar initial cDNA content of the sample. The results shown are representative of the three independent experiments. **(B)** KBM-5 cells (1 × 10^6^ cells/well) were treated with indicated concentrations of ART for 24 h. Thereafter, equal amounts of lysates were analyzed by Western blot analysis using antibodies against bcl-2, bcl-xL, survivin, and IAP-1/2. The same blots were stripped and reprobed with β-actin antibody to verify equal protein loading. **(C)** Cells (1 × 10^6^ cells/well) were treated with indicated concentrations of ART for 24 h. Total RNA was isolated, bax and p21 mRNA expressions were examined by RT-PCR analysis. GAPDH was performed to control for a similar initial cDNA content of the sample. The results shown are representative of the three independent experiments. **(D)** Cells (1 × 10^6^ cells/well) were treated with indicated concentrations of ART for 24 h. Thereafter, equal amounts of lysates were analyzed by Western blot analysis using antibodies against bax and p21. The same blots were stripped and reprobed with β-actin antibody to verify equal protein loading. **(E)** KBM-5 cells were treated with ART at 50 μM and 100 μM concentrations for 24 h and the cells were incubated with an FITC-conjugated Annexin V antibody and then analyzed by a flow cytometry. **(F)** After treatment of ART for 24 h, the cells were fixed and incubated using TUNEL reaction solution and then analyzed by a flow cytometry. **(G)** KBM-5 cells (1 × 10^4^ cells/well) were incubated at 37°C with 30 μM doxorubicin, 1.5 nM paclitaxel, and 1.5 nM docetaxel in the presence and absence of 10 μM ART as indicated for 24 h, and the viable cells were assayed using the MTT reagent. **(H)** KBM-5 cells (1 × 10^6^ cells/well) were treated 30 μM doxorubicin, 1.5 nM paclitaxel, and 1.5 nM docetaxel in the presence and absence of 10 μM ART as indicated for 24 h. Thereafter, equal amounts of lysates were analyzed by Western blot analysis using antibodies against survivin and PARP. The same blots were stripped and reprobed with β-actin antibody to verify equal protein loading.

Because bcl-2, bcl-xL, survivin, and IAP-1/2 have been implicated in tumor cell survival and mitochondrial dysfunction, we next examined the effects of ART on the constitutive expression of these mRNA and proteins. We found that ART substantially suppressed the expression of anti-apoptotic molecules both at protein and mRNA level in CML cells (Figure 3A and 3B).

### ART induces the expression of both bax and p21 in KBM-5 cells

The Bcl-2 family proteins have emerged as critical regulators of the mitochondria-mediated apoptosis by functioning as either promoters (e.g., bax and bak) or inhibitors (e.g., bcl-2 and bcl-xL) of the cell death process [40]. Once activated, bax permeabilizes the mitochondrial outer membrane, resulting in the release of cytochrome c and other pro-apoptotic factors that induce caspase activation and cell death [41]. Besides, the cyclin-dependent kinase inhibitor p21 is prototypical member of the Cip/Kip family of cyclin-dependent kinase inhibitors. It negatively modulates cell cycle progression by inhibiting the activities of cyclin E/CDK2 and cyclin D/CDK4 complexes and blocks DNA replication by binding to proliferating cell nuclear antigen [42]. We found that ART induced the expression of both bax and p21 at mRNA and protein levels in CML cells (Figure 3C and 3D).

### ART induces early and late apoptosis

To further demonstrate the anti-tumor effects of ART, we examined early apoptosis using the Annexin V antibody. The Annexin V positive cells (regarded as early apoptotic cells) were increased as compared with the non-treated cells as observed by flow cytometric analysis (Figure 3E). When we further examined for late apoptosis by analyzing DNA strand breaks using the TUNEL assay, percentage of cells undergoing apoptosis was also considerably increased upon ART treatment as observed by flow cytometric analysis (Figure 3F).

### ART potentiates the apoptotic effects of chemotherapeutic drugs

Paclitaxel and docetaxel (mitotic inhibitor) and doxorubicin (an anthracycline antibiotic) are major anticancer drugs used in the treatment of variety of cancers. To determine whether ART can also potentiate the apoptotic effect of these drugs, we treated KBM-5 cells with ART in combination with doxorubicin, paclitaxel, and docetaxel, and then examined the cell viability using a MTT assay. We found that ART indeed enhanced the cytotoxic effects of doxorubicin, paclitaxel, and docetaxel (Figure 3G). And then, to further confirm the potentiation effect of ART on chemotherapeutic drugs-induced apoptosis, we next determined whether the ART also down-regulates the expression of survivin, and induces PARP cleavage in KBM-5 cells by Western blot analysis when used in combination with these agents. As shown in Figure 3H, we found that combination treatment substantially enhanced the suppression of survivin expression and induced PARP cleavage as compared to the treatment with with individual drugs, indicating that ART can indeed potentiate chemotherapeutic drugs-induced apoptosis (Figure 3H).

### ART exhibits antitumor effects in a xenograft CML model

**Figure 4:**
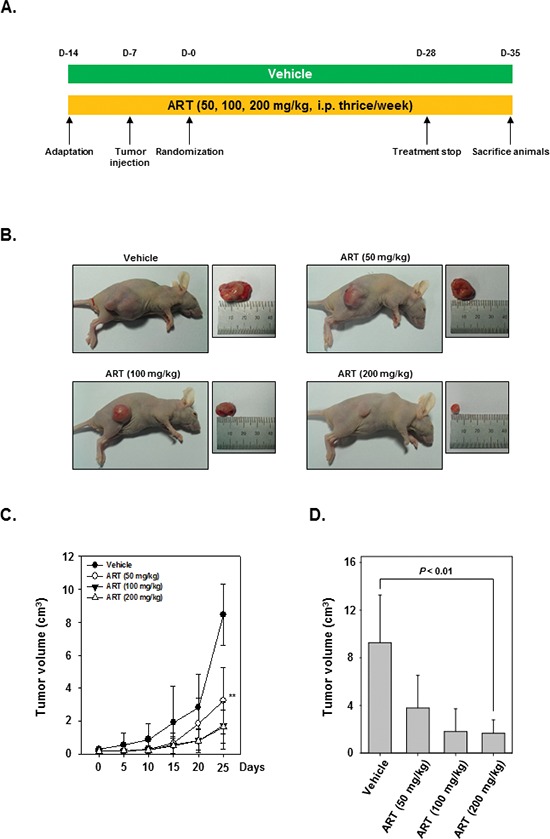
Effects of ART in human myeloid leukemia cells growth in nude mice induced by KBM-5 **(A)** Schematic representation of experimental protocol described in “Materials and Methods.” KBM-5 cells (4 × 10^6^ cells/mice) were injected subcutaneously into the right flank of the mice. The animals were randomized after 1 week of tumor cell injection into four groups based on tumor volume. Group I was given PBS (200 μL, i.p. thrice/week), group II was given ART (50 mg/kg body weight, i.p. thrice/week), group III was given ART (100 mg/kg body weight, i.p. thrice/week), and group IV was given ART (200 mg/kg body weight, i.p. thrice/week). **(B)** Necropsy photographs of mice bearing subcutaneously implanted myeloid leukemia tumors. **(C)** Tumor volumes in mice measured during the course of experiment and calculated using the formula V = 4 / 3 πr^3^, ** indicates *p* < 0.01. **(D)** Tumor volumes in mice measured on the last day of the experiment at autopsy using Vernier calipers and calculated using the formula V = 4 / 3 πr^3^ (*n* = 5). *Columns,* mean; *bars,* SE. ** indicates *p* < 0.01.

We examined the therapeutic potential of ART on the growth of subcutaneously implanted human CML KBM-5 cells in nude mice. The experimental protocol is depicted in Figure 4A. KBM-5 cells were implanted subcutaneously in the right flank of nude mice. When tumors have reached 0.25 cm in diameter after a week, the mice were randomized into 4 groups and started the treatment as per the experimental protocol. The tumor diameters were measured at 5-day intervals. The treatment was continued for 4 weeks and animals were sacrificed after 5 weeks. The tumors were excised and the tumor diameters were measured. We found that the tumor volume increased rapidly in the control group as compared to the other treatment groups (Figure 4B). Interestingly, we also noted that ART when given at 200 mg/kg body weight considerably inhibited the growth of the tumor at Day 25 after treatment (*P* < 0.01 when compared to control) (Figure 4C and 4D).

### ART downregulates the expression of the cell proliferation marker Ki-67

**Figure 5:**
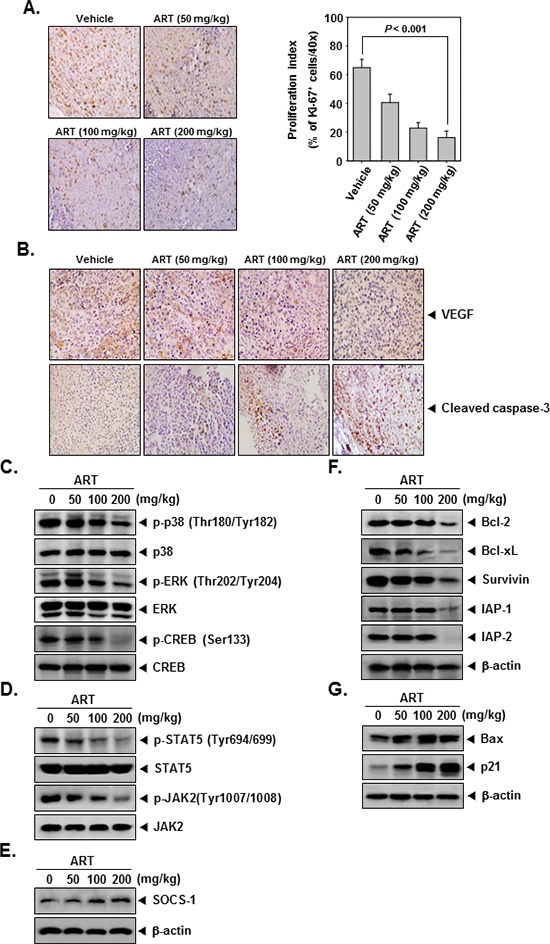
ART exerts the effect against tumor cell proliferation and angiogenesis in myeloid leukemia **(A)** Immunohistochemical analysis of proliferation marker Ki-67^+^ cell indicates the inhibition of human myeloid leukemia cells proliferation by ART dose-dependent treated groups of animals. Samples from 3 animals in each treatment group were analyzed, and representative data are shown (A, *left panel*). Quantification of Ki-67 proliferation index as described in “Materials and Methods.” Values are represented as mean ± SE of triplicate (A, *right panel*). *Columns,* mean of triplicate; *bars,* SE. **(B)** Immunohistochemical analysis of VEGF and cleaved caspase-3 in myeloid leukemia tumors. Samples from 3 animals in each treatment group were analyzed. **(C–E)** Western blot analysis showed the inhibition of p-p38, p-ERK, p-CREB, p-STAT5, p-JAK2, and SOCS-1 by ART in whole cell extracts from animal tissue. The same blots were stripped and reprobed with p38, ERK, CREB, STAT5, JAK2, and β-actin antibody to verify equal protein loading. **(F)** and **(G)** Equal amounts of lysates were analyzed by Western blot analysis using antibodies against bcl-2, bcl-xL, survivin, IAP-1, IAP-2, bax, and p21. β-actin was used as a loading control. Western blotting samples from three mice in each group were analyzed and representative data are shown.

To determine whether ART decreases myeloid leukemia tumor growth by inhibiting proliferation, we examined the expression of Ki-67^+^ cells in myeloid leukemia tumors from mice. Ki-67-positive index was used as a biomarker for cell proliferation. Our results showed that ART significantly decreased the expression of Ki-67 in tumor tissues in a dose-dependent manner (Figure 5A). (*P* < 0.001 vs. vehicle).

### ART downregulates the expression of VEGF

Because VEGF plays an important role in angiogenesis, we also examined its expression in CML tumors. We found that ART effectively suppressed the expression of VEGF in tumor tissues in a dose-dependent manner (Figure 5B, *upper panel*).

### ART activates caspase-3 in CML tumor tissues

To determine whether ART activates caspase-3, we examined the expression of cleaved caspase-3 in CML tumors from mice. Our results showed that ART increased the expression of caspase-3 and caused cleavage of caspase-3 in a dose-dependent manner (Figure 5B, *bottom panel* ).

### ART inhibits phosphorylation of p38, ERK, CREB, STAT5, and JAK2 in tumor tissues

We also evaluated the effect of ART on phosphorylation level of p38, ERK, CREB, STAT5, and JAK2 in CML tumor tissues. Figure 5C and 5D showed that ART was quite effective in suppressing the expression of p-p38, p-ERK, p-CREB, p-STAT5, and p-JAK2 in a concentration-dependent manner. ART had no effect on the expression of total p38, ERK, CREB, STAT5, and JAK2 proteins in tumor tissues (Figure 5C and 5D).

### ART induces the expression of SOCS-1 in tumor tissues

To determine whether ART induces the expression of SOCS-1, we examined the expression of this protein in myeloid leukemia tumors obtained from mice by Western blot analysis. Figure 5E shows that ART indeed induced the expression of SOCS-1 protein in a dose-dependent manner.

### ART down-regulates expression of various proteins involved in apoptosis in tumor tissues

We next examined using western blot analysis whether ART can modulate the constitutive expression of anti-apoptotic proteins in tumor tissues. We found that ART indeed suppressed the expression of bcl-2, bcl-xL, survivin, and IAP-1/2 in a dose-dependent manner (Figure 5F).

### ART induces the expression of both bax and p21 in tumor tissues

To determine whether ART induces the expression of bax and p21, we examined the expression of these proteins in myeloid leukemia tumors from mice by Western blot analysis. Figure 5G shows that ART induced the expression of both bax and p21 gene products in tumor tissues in a dose-dependent manner.

## DISCUSSION

The aim of this study was to determine whether ART can exert its anti-cancer effects in CML cells through targeted abrogation of diverse signal transduction cascades. This hypothesis was tested by using both a human phospho-antibody array system and xenograft mice model. We first found that this agent suppressed the phosphorylation of p38, ERK, CREB, Chk-2, STAT5, and RSK in human CML KBM-5 cells, also p53 protein became phosphorylated at Ser392 and Ser46, but not Ser15 in response to ART treatment. We further noted that ART can indeed suppress constitutive STAT5 and CREB activation, inhibit binding of STAT5 and CREB to the DNA, and reduce nuclear pool of STAT5 in KBM-5 cell with minimal effect on p38 and ERK activation. ART further down-regulated the expression of various gene products, including bcl-2, bcl-xl, survivin, IAP-1/2, while up-regulated bax and p21 gene expression in CML cells. Intraperitoneal injection of ART in a xenograft model of human CML KMB-5 cells resulted in a significant suppression of tumor progression and suppression of phosphorylation of p38/ERK/STAT5/CREB in ART-treated tumor tissues. These experimental results clearly indicate that ART acts upon multiple signal transduction cascades in CML.

We found for the first time that ART could suppress constitutive STAT5 activation in CML and that these effects were specific to STAT5 activation at Tyr694/699, as ART had no effect on the expression of total STAT5 protein. We also observed that ART suppressed nuclear translocation and DNA binding activity of STAT5. There is an abundant evidence to suggest that STAT5a and STAT5b are major player for lymphoid, myeloid, and erythroid cell development and function [43, 44]. Indeed, STAT5 proteins are activated by diverse cytokines, such as IL-2, IL-3, IL-5, IL-7, IL-9, IL-15, and erythropoietin [45–47]. Following cytokine stimulation, human STAT5a and STAT5b are phosphorylated on the conserved tyrosine residues Tyr-694 and Tyr-699, respectively, which allows for their dissociation from the receptor complex, formation of hetero or homodimers, and nuclear translocation to bind specific elements in the promoter of target genes and activate transcription [48]. Constitutive activation of STAT5 has been exhibited to be directly involved in oncogenic transformation [36]. STAT5 has been shown to be constitutively activated in lymphoid, myeloid, and erythroid leukemias [27–29], suggesting that ART may block oncogenesis through suppression of tyrosyl phosphorylation of STAT5.

How ART inhibits activation of STAT5 was investigated in detail. Recently, cross talk between the MAP kinase pathways and the activation of the different STAT transcription factors has been described. MAPK kinase (MEK) inhibitor has been shown to suppress growth hormone-induced transcription mediated by STAT5, which can also directly interact with ERK1/2 [24, 25]. We observed that ART inhibited the phosphorylation of p38 and ERK using both human phospho-antibody array system and Western blot analysis. Our data suggests that ART could possibly affect the interaction between the mitogen-activated protein kinase (MAPK) and STAT5 pathways. Additionally, we found for the first time that ART could suppress phosphorylation of CREB and also its DNA binding activity. Recent studies have shown that CREB is involved tumor initiation, progression and metastasis, thereby supporting its role as a proto-oncogene [49]. Shankar *et al* have previously demonstrated that CREB overexpression is also linked with increased risk of relapse and decreased event-free survival in acute myeloid leukemia [32]. Overall, it was found that ART could suppress phosphorylation of multiple proteins (p38/ERK/STAT5/CREB) as confirmed through the corroboration between the phospho array system and Western blot analysis.

We further found that the expression of several anti-apoptotic gene products (e.g. bcl-2, bcl-xl, survivin) was suppressed by ART. Constitutively active STAT5/CREB is closely associated with oncogenesis by preventing cancer cells from apoptosis [27, 29, 49]; this implies that suppression of the transcriptional factors by ART could facilitate apoptosis. ART has been shown to induce apoptosis, activate caspase-3 and increase the Bax/Bcl-2 ratio and poly (ADP-ribose) polymerase in both human hepatoma cells [13] multiple myeloma and diffuse large B-cell lymphoma (DLBCL) [50]. The survivin expression is reported to be increased by STAT5 in T cell leukemia [51]. Bcl-2 and Bcl-xL can also block cell death induced by a variety of chemotherapeutic drugs, and thus contribute to chemoresistance [52]. It has been previously reported that ART selectively down-regulates survivin that contributes to a radio-sensitization of glioma cells by an increased induction of apoptosis [53]. We also observed that ART substantially potentiated the apoptotic effect of doxorubicin, paclitaxel, and docetaxel in KBM-5 cells and hence could also be used in conjunction with existing anti-CML therapies.

We further noted that ART significantly suppressed CML growth in a xenograft mouse model down-regulated the expression of phospho-p38/ERK/STAT5/CREB and increased the levels of caspase-3 in treated group as compared with control. The down-modulation of VEGF expression in tumor tissues by ART also emphasized its anti-angiogenic potential in CML, an aspect which requires further investigations. Overall, our experimental observations clearly indicate that the anti-cancer effects of ART in human CML cells are mediated through the suppression of diverse signal transduction cascades and provide a strong rationale for pursuing the use of ART to enhance treatment efficacy in CML patients.

## MATERIALS AND METHODS

### Reagents

Artesunate (ART), 3-(4,5-dimethylthiazol-2-yl)-2,5-diphenyltetrazolium bromide (MTT), propidium iodide (PI), Tris base, glycine, NaCl, sodium dodecyl sulfate (SDS), RNase A, DPX mountant for histology, and bovine serum albumin (BSA) were purchased from Sigma-Aldrich (St. Louis, MO). Iscove Modified Dulbecco Medium (IMDM), RPMI 1640, and fetal bovine serum (FBS) were obtained from Lonza Group Ltd. (Basel, Switzerland). 0.4% Trypan Blue solution, and antibiotic-antimycotic mixture was obtained from Life Technologies (Grand Island, NY). Anti-phospho-p38, anti-p38, anti-phospho-ERK, anti-ERK, anti-phospho-CREB, anti-CREB, anti-phospho-JAK2, anti-JAK2, anti-procaspase-3, and anti-cleaved caspase-3 antibodies were purchased from Cell Signaling Technology (Beverly, MA). Anti-phospho-STAT5, anti-STAT5, anti-SOCS-1, SOCS-1 siRNA, anti-bcl-2, anti-bcl-xL, anti-survivin, anti-cyclin D1, anti-IAP-1, anti-IAP-2, anti-PARP, anti-Ki-67, anti-VEGF, anti-β-actin, and horseradish peroxidase (HRP)-conjugated secondary antibodies were obtained from Santa Cruz Biotechnology (Santa Cruz, CA). TUNEL (terminal transferase mediated dUTP-fluorescein nick end labeling) assay kit was from Roche Diagnostics GmbH (Mannheim, Germany). Whole-cell lysates of tumor tissues were obtained with T-PER Tissue Protein Extraction Reagent (Pierce, Rockford, USA).

### Cell lines

Human myeloid leukemia KBM-5, human multiple myeloma cell lines such as U266 and MM1.S (melphalan-sensitive), human pancreas adenocarcinoma AsPC-1, human lung carcinoma H1299, human prostate carcinoma DU145, and human breast carcinoma MCF-7 cells were obtained from the American Type Culture Collection (Manassas, VA). KBM-5 cells were cultured in IMDM medium supplemented with 15% FBS. All other cells were cultured in RPMI 1640 medium containing 10% FBS. All media were also supplemented with 100 U/ml of penicillin and 100 μg/ml of streptomycin.

### Human phospho-kinase array

For antibody arrays three hundred micrograms of cellular extracts were incubated with the Human Phospho-Kinase Array Kit (Proteome Profiler™; R&D Systems, Minneapolis, MN) following manufacturer's instructions. Densitometry values for Western blot and antibody array experiments were estimated by the Image J software (National Institutes of Health, Maryland, U.S.) and were expressed as arbitrary units (a.u.). Multiple film exposures were used to verify the linearity of the samples analyzed and to avoid saturation of the film. In antibody arrays, the average signal of the pair of duplicate spots, representing each phosphorylated kinase protein, was calculated after subtraction of background values (pixel density) from negative control spots and normalization to average values from positive control spots.

### Western blotting

After the cells were treated with the indicated concentrations of ART, the cells were lysed and the total protein concentrations were determined by Bradford reagent (Bio-Rad, Hercules, CA). Equal amounts of lysates resolved on sodium dodecyl-polyacrylamide gel electrophoresis (SDS-PAGE) were transferred to a nitrocellulose membrane, and the membrane was blocked with 1× TBS containing 0.1% Tween 20 and 5% skimmed milk or 2% BSA for 1 h at room temperature. After the blocking, the membranes were incubated overnight at 4°C with the respective primary antibodies. The membranes were washed twice and incubated with diluted horseradish peroxidase (HRP)-conjugated secondary antibodies (1:10000) for 1 h at room temperature. After three washes, the membranes were detected using an enhanced chemiluminescence (ECL) kit (GE Healthcare, Waukesha, USA).

### Electrophoretic mobility shift assay (EMSA)

STAT5 and CREB-DNA binding was analyzed by EMSA using a ^32^P-labeled high-affinity cis-inducible element (hSIE) probe. The sequences of STAT5 DNA-binding elements was 5′-AGA TTT CTA GGA ATT CAA TCC-3′ and CREB DNA-binding elements was 5′-AGA GAT TGC CTG ACG TCA GAG AGC TAG-3′ (Santa Cruz Biotechnology, Santa Cruz, CA). Briefly, nuclear extracts were prepared and incubated with the labeled hSIE probe. The DNA-protein complex formed was separated from free oligonucleotide on 5% native polyacrylamide gels. The dried gels were visualized with an Universial hood II (Bio-rad, Hercules, CA).

### Transfection with SOCS-1 siRNA

We investigated the ability of commercially available electroporation systems, the Neon™ Transfection System (Invitrogen, Carlsbad, CA). Transfection efficiency was measured by Western blot analysis. KBM-5 cells were prepared for transfection, after cells were resuspended with 120 μl of Neon Resuspension Buffer R for every one million cells. For each electroporation, KBM-5 cells with 50 nM of SOCS-1 siRNA (Santa Cruz, CA) were aliquoted into a sterile microcentrifuge tube. A Neon Tip was inserted into the Neon Pipette and the cell-siRNA mixture was aspirated into the tip avoiding air bubbles. The Neon Pipette was then inserted into the Neon Tube containing 3 ml of Neon Electrolytic Buffer E in the Neon Pipette Station. Cells were pulsed once with a voltage of 1,300 and a width of 20 ms. After 48 h of transfection, cells were treated with 100 μM of ART for 4 h, and whole-cell extracts were washed twice with ice-cold PBS, lysed with lysis solution, and cell lysates were prepared for Western blot analysis.

### Immunocytochemistry for STAT5 localization

After the KBM-5 cells were treated with the 100 μM of ART, the cells suspension was put into the cytospin and the assembly was placed into the rotor. Thereafter it was centrifuged for 5 min at 750 rpm. The cells were attached the slide and analyze under the microscope whether monolayer spread of cells obtained. The cells were fixed in 4% paraformaldehyde (PFA) for 20 min at room temperature and then washed three times in PBS. The cells were permeabilized with 0.2% Triton X-100 in PBS for 20 min, washed three times in PBS, and then blocked with 5% normal goat serum in PBS for 1 h at room temperature. The cells were then incubated overnight at 4°C with anti-STAT5 (1:100 dilution), washed three times, and incubated with FITC-conjugated secondary antibodies (1:200; Jackson Immuno Research, West Grove, PA) for 1 h at room temperature. Next, the cells were stained with a 1 μg/ml DAPI solution and mounted on glass slides using CRYSTAL/MOUNT™ (Biomeda Corp., Foster City, CA). Using an Olympus FluoView FV1000 confocal microscope (Tokyo, Japan), DAPI and FITC fluorescence were excited (Ex: 405 nm and 488 nm) and detected (Em: 461 nm and 519 nm) with 2.1% laser transmissivity and 5.0% laser transmissivity respectively.

### Cell cycle analysis

Cell cycle analysis was performed using PI. After treatment with ART, the cells were collected, washed with cold PBS, fixed with 70% ethanol, and incubated for 30 min at 37°C with 0.1% RNaseA in PBS. Cells were then washed, resuspended, and stained in PBS containing 25 μg/ml of PI for 30 min at room temperature. Cell distribution across the cell cycle was analyzed with a flow cytometry (Becton–Dickinson, Heidelberg, Germany).

### MTT assay

Cell viability was measured by an MTT assay to detect NADH-dependent dehydrogenase activity. Fifty microliters of MTT solution (5 mg/ml) in 1× phosphate-buffered saline (PBS) was directly added to the cells, which were then incubated for 4 h to allow MTT to metabolize to formazan. Absorbance was measured with an automated spectrophotometric plate reader at a wavelength of 570 nm. Cell viability was normalized as relative percentages in comparison with untreated controls.

### RNA analysis and reverse transcription-PCR

KBM-5 cells were treated with the indicated concentrations of ART, washed, and suspended in Trizol reagent. Total RNA was extracted according to the manufacturer's instructions (Invitrogen, Life Technologies). Total RNA was reverse transcribed into cDNA using an oligo (dT)_15_ premix (Intron, Korea). The relative expression of *socs-1, bcl-2*, *bcl-xL*, *survivin*, *bax*, and *p21* was analyzed using quantitative RT-PCR with glyceraldehyde-3-phosphate dehydrogenase (GAPDH) as an internal control. The following pairs of forward and reverse primer sets were used: socs-1, 5′-ACGCAGCATTAACTGGGATG-3′ and 5′-CCCTGGTTTGTGCAAAGATACT-3′. bcl-2, 5′-TTGTGGCCTTCTTTGAGTTCGGTG-3′ and 5′-TACAGTTCCACAAAGGCATCCCAG-3′. bcl-xL, 5′-TACCAGCCTGACCAATATGGCGAA-3′ and 5′-TGGGTTCAAGTGATTCTCCTGCCT-3′. survivin, 5′-ATGGGTGCCCCGACGTT-3′ and 5′-TCAATCCATGGCAGCCAG-3′. bax, 5′-GAGAGGTCTTTTTCCGAGTGG-3′ and 5′-CCTTGAGCACCAGTTTGCTG-3′. p21, 5′-AGGTCTTGGATTGAGGAACAG-3′ and 5′-TTTGCAGCAGACAACAATGGCT-3′. PCR products were run on 1% agarose gel and then stained with Loading Star (Dynebio, Korea). Stained bands were visualized under UV light and photographed.

### Annexin V assay

One of the early indicators of apoptosis is the rapid translocation and accumulation of the membrane phospholipid phosphatidylserine from the cell's cytoplasmic interface to the extracellular surface. This loss of membrane asymmetry can be detected using the binding properties of annexin V. To detect apoptosis, we used annexin V antibody conjugated with the fluorescent dye fluorescein isothiocyanate (FITC). KBM-5 (1 × 10^6^ cells/well) cells were treated with ART for 24 h, and then stained by Annexin V conjugated to FITC. The cells were washed and observed accordingly with a flow cytometry (Becton–Dickinson, Heidelberg, Germany).

### TUNEL assay

After treatment with ART for 24 h, cells were fixed in 4% paraformaldehyde at room temperature for 1 h, washed with PBS and permeabilized in 0.1% sodium citrate, containing 0.1% Triton X-100, at 4°C for 20 min. Cells were then resuspended in a final volume of 25 μl of TUNEL reaction mixture (2.5 μl TUNEL-Enzyme in 22.5 μl TUNEL Label, Roche), with the addition of 20 mM EDTA and then counterstained with 5 μg/ml propidium iodide in PBS containing 0.5 μg/ml DNase-free RNaseA, for 1 h at 37°C in a humidified atmosphere in the dark. After being washed with PBS, cells were analyzed by a flow cytometer (Becton–Dickinson, Heidelberg, Germany). Acquisition and analysis of the data was performed using Cell Quest 3.0 software.

### Animals

All procedures involving animals were reviewed and approved by KHU Institutional Animal Care and Use Committee [KHUASP(SE)-12–038]. Six week-old athymic nu/nu female mice (NARA Biotech, Korea) were implanted subcutaneously in the right flank with KBM-5 cells. The animals were housed (6 mice/cage) in the standard mice plexi glass cages in a room maintained at constant temperature and humidity under 12 h light and dark cycle and fed with regular autoclaved mouse chow with water *ad libitum*. None of the mice exhibited any lesions and all were tested pathogen-free. Before initiating the experiment, we acclimatized all mice to a pulverized diet for 3 days.

### Subcutaneous implantation of KBM-5 cells

KBM-5 cells were harvested from subconfluent cultures, washed once in serum-free medium, and resuspended in PBS. Only suspensions consisting of single cells, with > 90% viability, were used for the injections. KBM-5 cells [4 × 10^6^/100 μL PBS:Matrigel (1:1)] were injected subcutaneously into the left flank of the mice. To prevent leakage, a cotton swab was held cautiously for 1 minute over the site of injection.

### Experimental protocol

When tumors have reached 0.25 cm in diameter, the mice were randomized into the following treatment groups (*n* = 6/group). Group I was given PBS (200 μL, i.p. thrice/week), group II was given ART (50 mg/kg body weight, i.p. thrice/week), group III was given ART (100 mg/kg body weight, i.p. thrice/week), and group IV was given ART (200 mg/kg body weight, i.p. thrice/week). Therapy was continued for 4 weeks, and the animals were euthanized 1 week later. Primary tumors were excised and the final tumor volume was measured as V = 4 / 3 πr^3^, where r is the mean radius of the three dimensions (length, width, and depth). Half of the tumor tissue was fixed in formalin and embedded in paraffin for immunohistochemistry and routine hematoxylin and eosin (H&E) staining. The other half was snap frozen in liquid nitrogen and stored at –80°C.

### Western blot analysis for tumor tissues

Myeloid leukemia tumor tissues (75–100 mg) from control and experimental mice were minced and incubated on ice for 30 minutes in 0.5 ml of ice-cold T-PER Tissue Protein Extraction Reagent (Pierce, Rockford, USA). The minced tissue was centrifuged at 16,000 × g at 4°C for 20 minutes. The proteins were then fractionated by SDS-PAGE, electrotransferred to nitrocellulose membranes, blotted with each antibody, and detected by enhanced chemiluminescence (ECL) kit (GE Healthcare, Waukesha, USA).

### Immunohistochemical analysis of myeloid leukemia tumor samples

Solid tumors from control and various treatment groups were fixed with 10% phosphate buffered formalin, processed and embedded in paraffin. Sections were cut and deparaffinized in xylene, and dehydrated in graded alcohol and finally hydrated in water. Antigen retrieval was performed by boiling the slide in 10 mM sodium citrate (pH 6.0) for 30 min. Immunohistochemistry was performed following manufacturer instructions (Vector Laboratories ImmPRESS™ REAGENT KIT). Briefly, endogenous peroxidases were quenched with 3% hydrogen peroxide. Non-specific binding was blocked by incubation in the blocking reagent in the ImmPRESS™ REAGENT KIT (Vector Laboratories, Burlingame, CA) according to the manufacturer's instructions. Sections were incubated overnight with primary antibodies as follows: anti-Ki-67, anti-cleaved caspase-3, and anti-VEGF (each at 1:100 dilutions). Slides were subsequently washed several times in phosphate-buffered saline (PBS) and were incubated with ImmPRESS™ reagent according to the manufacturer's instructions. Immunoreactive species were detected using 3, 3-diaminobenzidine tetrahydrochloride (DAB) as a substrate. Sections were counterstained with Gill's hematoxylin and mounted under glass coverslips. Images were taken using an Olympus BX51 microscope (magnification, 20×). Positive cells (brown) were quantitated using the Image-Pro plus 6.0 software package (Media Cybernetics, Inc.).

### Statistical analysis

All numeric values are represented as the mean ± SD. Statistical significance of the data compared with the untreated control was determined using the Student unpaired *t*-test. Significance was set at *P* < 0.05.
